# Association between posttraumatic stress disorder (PTSD) severity and ego structure of the Nanai people

**DOI:** 10.1186/s12199-017-0666-z

**Published:** 2017-07-10

**Authors:** Yoko Ota, Natalia Korshunova, Masashi Demura, Midori Katsuyama, Hironobu Katsuyama, Sri Ratna Rahayu, Kiyofumi Saijoh

**Affiliations:** 1 Department of Hygiene, School of Medicine, Kanazawa University, Kanazawa, Japan; 2 Department of Public Health, Kawasaki Medical University, Kurashiki, Japan

**Keywords:** Post-traumatic Stress Disorder, Ego structure Test by Ammon, A man-made chemical disaster, indigenous Nanai people

## Abstract

**Background:**

A man-made chemical disaster occurred in the Amur River, leading to posttraumatic stress disorder (PTSD) in the Nanai people indigenous to the river’s surrounding area. PTSD severity measured by the total scores of Impact of Event Scale-Revised (IES-R) (Total-I) and Clinician-Administered PTSD Scale (CAPS) (Total-C) were not always identical in terms of demographic and ethnocultural characters. It is possible that the results derived using the Total-I and Total-C may differ for persons with different backgrounds and/or individual characteristics. In this study, the associations between PTSD severity and personal characteristics were evaluated.

**Methods:**

The study was a field-type survey including 187 randomly selected participants (75 males and 112 females). In addition to Total-I/Total-C, scores for each IES-R/CAPS item, Intrusion, Avoidance, and Hyperarousal, and Ego Structure Test by Ammon (ISTA) score were examined to evaluate their personal characteristics.

**Results:**

No specific trends in ISTA score were obvious among four groups defined according to Total-I/Total-C. The results of principal component analysis showed that all IES-R/CAPS items contributed positively to the 1st axis but to the 2nd axis in a different manner. ISTA items did not always show correlations to each other, but principal component analysis suggested that Construct contributed positively and Destruct and Deficient (with the exception of Destruct sexuality) contributed negatively. High IES-R scores were associated with Construct Aggression and Deficient Inner demarcation, but high CAPS score was less likely to exhibit Construct Narcissism.

**Conclusion:**

To avoid the misdiagnosis of PTSD, usage of both IES-R/CAPS may be required. Simultaneous application of personality/ego tests may be helpful, but appropriate numbers of their questions would be important.

## Background

In December 2005, an accident at a chemical factory caused the release of toxic substances into the Songhua River (Jilin, China); these substances then polluted the downstream Amur River, which lies in the Russian territory [1]. The serious subsequent pollution of the river water caused several adverse outcomes, including the sedimentation of chemicals into the riverbed, forest fires [2], the prohibition of fishing and hunting, and a reduction in the forest area. Many of the Nanai people that lived along the middle reaches of the Amur River Valley lost their traditional ways of obtaining staple food and performing their traditional and religious activities and suffered from posttraumatic stress disorder (PTSD).

Previously [3], PTSD severity has been assessed in this population using the Impact of Event Scale-Revised (IES-R) [4–6] and the Clinician-Administered PTSD Scale (CAPS) [7, 8]. Both the IES-R and the CAPS consist of questions regarding Intrusion (compulsion to repeat), Avoidance of traumatic events, and Hyperarousal to physiological symptoms of irritability. Severity was determined by assessing their total scores, IES-R total (Total-I) and CAPS total (Total-C). The results were not always identical and depended upon the participants’ demographic and ethnocultural background, clinical examination results, and ethnopsychological attitudes toward the Amur River [3]. The comparison of the results derived using the Total-I and the Total-C alone seemed to be insufficient to understand the severity of PTSD in this population. Moreover, interactions between personal characteristics differentially affected on Intrusion, Avoidance, and Hyperarousal may have existed. In fact, when attempting to obtain a better understanding of PTSD severity, the effects of an individual’s personality profile on his or her PTSD severity were of interest in various situations [9–12]. In the present study, the Ego Structure Test by Ammon (ISTA) [13] was utilized to gain a better understanding of the participants’ personality profiles, because it was available in Russian, and we analyzed the interactions between ISTA, IES-R and CAPS items, and PTSD severity.

## Methods

### Subjects

The participants and field-type survey have been described previously [3]. That is, 187 indigenous adult Nanai volunteers over the age of 18 years were randomly selected from the general civilian population in eight villages in the Nanai Regional District of Khabarovsk Territory located in the Far East of the Russian Federation. The study design was approved by the Ethical Committee of Kanazawa University School of Medicine (Japan) and the Ethical Committee of Far Eastern State Medical University (Russian Federation). This study’s participants belonged to an ingenious population with its own customs and religion. All consent forms were signed by village patriarchs.

### Survey and study design

Study subjects were recruited for field survey participation by visiting the yards of eligible participants living in residential areas during the daytime (usually from 9 a.m. to 6 p.m.). The survey was carried out during the ecological catastrophe in winter and spring 2006. Two medical doctors trained in the specifics of PTSD research conducted the interviews under the supervision of a senior interviewer. The questionnaires were assigned ID numbers to protect the identities of the participants. PTSD severity was assessed using the Russian-validated versions [6] of the IES-R [4, 5] and CAPS [7, 8], and the participants’ personality characteristics were measured using the ISTA [13].

PTSD severity, as determined via the participants’ Total-I and Total-C, was analyzed in association with the participants’ demographic and ethnocultural background, clinical examination results, and ethnopsychological attitude toward the Amur River; these data have been previously published [3]. Due to their discrepancy identified in the derived results, we proceeded to analyze the association between the participants’ personality/ego profiles and not only Total-I and Total-C but also specific items on the IES-R and CAPS.

PTSD examination: the precise method utilized for IES-R and CAPS administration has been described in a previous report [3]. Briefly, they are questionnaires consisted of four to eight questions scoring severity of Intrusion/Avoidance/Hyperarousal and their total.

Personality profile measurement using the ISTA: The ISTA is also a questionnaire, which is somewhat uncommon in Western countries, but a Russian form is available [14]. We aimed to clarify personality/ego structure using ISTA questionnaires divided into categories that included Aggression, Anxiety/Fear, Outer ego demarcation, Inner ego demarcation, Narcissism, and Sexuality. Some questions of ISTA might relate to IES-R and CAPS questions, whereas each category was estimated by 11 to 14 yes/no questions to judge their constructiveness (Construct), destructiveness (Destruct), and deficiency (Deficient) [13, 14]. Thus, personality/ego structures judged by ISTA are clearly different from those by IES-R/CAPS, and their effects on PTSD severity judged by IES-R/CAPS were targeted to analyze in the present study.

### Statistical analysis

Several cutoff points have been reported as follows: for Total-I, scores ≥25 indicate persons at high risk of PTSD [15, 16] and scores ≥34 indicate probable or confirmed PTSD cases [15, 16]; additionally, for Total-C, scores of 20–39 indicate mild PTSD [17] and scores of 40–59 indicate moderate PTSD [17]. To separate the participants into four groups, cutoff scores of 34 (for the Total-I) and 40 (for the Total-C) were used as cutoffs in the present study; individuals with low Total-I scores (<34) and low Total-C scores (<40) were categorized into the LL group; those with low Total-I scores (<34) and high Total-C scores (≥40) were categorized into the LH group; those with high Total-I scores (≥34) and low Total-C scores (<40) were categorized into the HL group, and those with high Total-I scores (≥34) and high Total-C scores (≥40) were categorized into the HH group. Members of the LL group were considered to potentially not have PTSD; members of the HH group were considered to possibly have PTSD; and in the LH and HL groups, the diagnosis of PTSD was ambiguous. The ISTA scores in each group were evaluated using one-way ANOVA tests with Tukey’s honest significant difference (HSD) employed as a post hoc test. To clarify the characteristics of the IES-R and CAPS items, principal component analysis was applied. For ISTA items, after a correlation matrix was made, principal component analysis was performed. Thereafter, IES-R and CAPS items were analyzed by stepwise regression analysis using ISTA items as determinants. All statistical analyses were made using JMP11 (SAS Inst. Inc, Cary, NC, USA).

## Results

### Analyses of ISTA scores in groups defined by participant Total-I and Total-C scores

The demographic and ethnocultural characteristics of the included subjects and their effects on the participants’ Total-I and Total-C scores have been described elsewhere [3]. The correlation between the Total-I and Total-C scores identified in this population was not always high (*r* = 0.45, [3]); hence, the participants were categorized into four groups: LL (*n* = 77), LH (*n* = 29), HL (*n* = 43), and HH (*n* = 38) (Table 1), and ISTA scores were compared between the aforementioned groups. The participants’ Destruct and Deficiency scores tended to be lower in the LL group (Table 1). However, significantly higher values were observed in the following comparisons: Construct Aggression (HL vs. LH), Anxiety (HL vs. LL), and Sexuality (HL vs. HH), as well as total score (HL vs. LL) and Destruct Anxiety (LH and HH vs. LL). Additionally, all HH groups had higher Deficiency scores than did the LL groups. Higher scores were also identified in the following comparisons: Deficiency Aggression (HH vs. LH), Inner demarcation (HH vs. LH and HL), Narcissism (LH and HH vs. HL), Sexuality (HH vs. LH and HL), and total score (LH vs. LL and HH vs. HL). The comparison between average ISTA item scores seemed to be insufficient to distinguish between the LH and HL groups, i.e., whether LH and/or HL had PTSD or not remained poorly understood.

**Table 1:** Differences in the ISTA scores among groups defined by Total-I/Total-C

		Construct				Destruct				Deficiency		
	LL	LH	HL	HH	LL	LH	HL	HH	LL	LH	HL	HH
Aggression	8.2 ± 2.5	7.7 ± 2.6	9.4 ± 2.0^b^	8.3 ± 2.2	4.8 ± 2.7	6.3 ± 2.2	6.0 ± 2.8	6.6 ± 3.2^a^	3.4 ± 1.9	4.1 ± 2.2	4.4 ± 2.1	5.5 ± 2.4^a, b^
Anxiety	7.2 ± 2.4	8.2 ± 2.0	8.4 ± 2.2^a^	8.3 ± 2.3	2.1 ± 2.0	3.6 ± 2.4^a^	2.6 ± 2.2	4.0 ± 2.1^a, c^	3.3 ± 1.8	4.2 ± 2.5	3.7 ± 2.3	4.8 ± 2.4^a^
Outer demarcation	8.2 ± 1.7	8.3 ± 1.5	8.5 ± 1.6	7.9 ± 1.7	4.9 ± 2.0	5.7 ± 1.7	5.1 ± 1.8	5.8 ± 1.9^a^	4.2 ± 2.0	5.3 ± 2.8	5.4 ± 2.8^a^	6.0 ± 2.2^a^
Inner demarcation	9.4 ± 1.9	9.3 ± 1.8	9.9 ± 1.6	9.3 ± 1.9	4.1 ± 2.1	5.0 ± 1.8	4.6 ± 1.8	5.4 ± 1.9	4.2 ± 2.0	5.7 ± 2.8	5.4 ± 2.8	7.6 ± 2.5^a, b, c^
Narcissism	7.9 ± 2.3	7.4 ± 2.3	8.8 ± 2.2	7.5 ± 2.3	3.6 ± 2.1	3.8 ± 2.1	4.2 ± 1.9	5.1 ± 2.2^a^	3.0 ± 2.3	4.6 ± 2.5a	3.0 ± 2.4^b^	4.4 ± 2.3^a, c^
Sexuality	7.0 ± 3.8	6.9 ± 3.6	8.1 ± 3.5	5.7 ± 4.0 ^c^	2.8 ± 2.3	4.1 ± 3.1	3.8 ± 2.7	3.7 ± 2.7	2.3 ± 1.7	2.7 ± 2.2	2.9 ± 2.2	4.2 ± 2.7^a, b, c^
Total	48.0 ± 11.0	47.9 ± 10.5	53.1 ± 9.1	47.1 ± 9.4^c^	22.3 ± 7.9	28.5 ± 8.0	26.4 ± 7.3	30.5 ± 9.5^a^	20.3 ± 8.1	26.7 ± 9.3^a^	24.8 ± 9.9	32.4 ± 10.3^a, c^

Subjects were grouped according to Total-I/Total-C; cutoff is 34 for Total-I and 40 for Total-C, respectively. The group was defineded as L if lower than cutoff and H if equal or higher than cutoff. Number: LL 77, LH 29, HL 43, and HH 38

Significant differences (^a^vs. LL, ^b^vs. LH, and ^c^vs. HL; *P* < 0.05, one-way ANOVA followed by Tukey’s HSD as post hoc)

### Principal component analysis of IES-R and CAPS scores

**Fig. 1:**
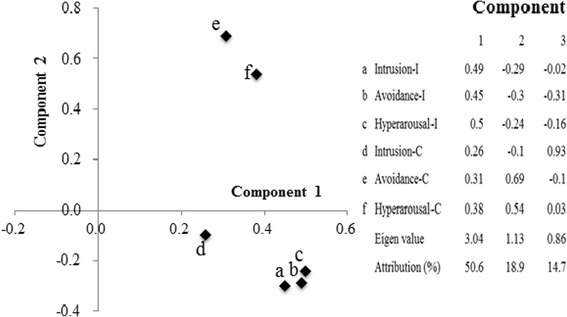
The principal component analysis of Intrusion-I, Avoidance-I, Hyperarousal-I, Intrusion-C, Avoidance-C, and Hyperarousal-C. *a* Comparison of components *1* and *2. b* Values of components *1*–*3*. Statistically significant eigenvectors are indicated in *bold* (*P* < 0.05)

To clarify the reasons for the differences identified in Total-I and Total-C, a principal component analysis was performed on each IES-R and CAPS item. All IES-R and CAPS scores contributed positively to component 1 (Fig. 1). Intrusion-I/Avoidance-I/Hyperarousal-I contributed negatively and Avoidance-C/Hyperarousal-C contributed positively to component 2. Moreover, component 3 consisted of negative Avoidance-I/Hyperarousal-I and positive Intrusion-C. The attribution rates of these three components were 50.6, 18.9, and 14.7%, respectively, and the cumulative attribution rate was 84.2%.

### Correlation matrix and principal component analysis of ISTA scores

**Fig. 2:**
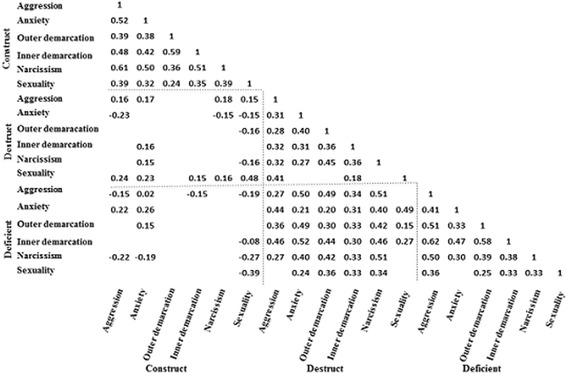
Correlation matrix of ISTA scores. Only significant (*P* < 0.05) values are presented

According to the correlation matrix, constructive items were positively correlated with each other (Fig. 2). Construct Anxiety was positively correlated with several Destruct and Deficiency items, and Construct Aggression and Sexuality were also somewhat correlated; however, these correlations were sometimes negative. The Destruct and Deficient items were usually positively correlated with one another. As expected based on these results, Construct was separated from Destruct and Deficient within the principal component analysis. Component 1 consisted of all Destruct and Deficient items excluding Destruct Sexuality, and component 1 had an attribution rate of 27.1% (Table 2). Component 2 comprised all Construct items and Destruct Sexuality, and its attribution rate was 20.0%. Hence, the cumulative attribution for these two components was 47.1%. All components until component 10 were determined to be significant, and the attribution rate reached 84.5%; however, the eigenvalue of each component was not always as high as those of components 1 and 2 (data not shown).

**Table 2:** Principal component analysis of ISTA scores

Eigenvectors	Component	
	1	2
Construct		
Aggression		0.41
Anxiety		0.38
Outer demarcation		0.31
Inner demarcation		0.37
Narcissism		0.40
Sexuality		0.35
Destruct		
Aggression	0.26	
Anxiety	0.29	
Outer demarcation	0.29	
Inner demarcation	0.26	
Narcissism	0.32	
Sexuality		0.25
Deficient		
Aggression	0.36	
Anxiety	0.27	
Outer demarcation	0.32	
Inner demarcation	0.36	
Narcissism	0.30	
Sexuality	0.22	
Eigenvalue	4.87	3.61
Attribution (%)	27.1	20.0

Components 1–10 were judged to be statistically significant and covered 84.5% in total. However, excluding components 1 and 2, the attribution of each component was small; hence, components 1 and 2 alone were indicated. Significant contribution to eigenvalue alone was listed

### Stepwise regression of the associations between IES-R and CAPS items and ISTA items

In the stepwise regression analysis of the associations between IES-R and CAPS items toward ISTA items (used as determinants), Deficient Inner demarcation was included in Intrusion-I/Avoidance-I/Hyperarousal-I, and Construct Aggression was included in Intrusion-I/Avoidance-I; therefore, these items were included in Total-I (Table 3). Destruct Anxiety was identified in Avoidance-I but also appeared in Total-I; however, Deficient Sexuality was identified in Intrusion-I and Destruct Narcissism in Hyperarousal-I; thus, these items did not appear in Total-I. No significant determinants of Intrusion-C inclusion were identified. The presence of Negative Construct Narcissism in Avoidance-C/Hyperarousal-C was associated with Construct Narcissism in Total-C. Deficient Inner demarcation in Avoidance-C and Construct Anxiety in Hyperarousal-C also appeared in Total-C. Destruct Anxiety appeared in Hyperarousal-C but not in Total-C.

**Table 3:** Stepwise regression of IES-R and CAPS scores using ISTA scores as determinants

	IES-R				CAPS			
	Intrusion	Avoidance	Hyperarousal	Total-I	Intrusion	Avoidance	Hyperarousal	Total-C
Construct								
Aggression	0.57	0.85		1.92				
Anxiety							0.89	1.65
Narcissism						−0.65	−0.61	−1.64
Destruct								
Anxiety		0.83		1.99			1.11	
Narcissism			0.64					1.25
Deficient								
Inner demarcation	0.58	0.50	0.72	1.68		0.95		1.41
Sexually	0.60							

ISTA items were listed when stepwise regression analysis was extracted (*P* < 0.05) as factors explaining IES-R/CAPS scores. Values are expressed as standardized partial regression coefficient

## Discussion

Both the IES-R and CAPS have not only been used in various epidemiological studies to assess the prevalence of PTSD but have also been used to estimate the frequency and intensity of individual symptoms and disorders by facilitating the screening and quick assessment of patient status [18, 19]. For such purposes, Total-I and Total-C, calculated as the sum of scores of three questionnaire categories (Intrusion, Avoidance, and Hyperarousal), are usually used. These categories are highly correlated but considered to have some discrepancies when used for the assessment of PTSD [20–24]. Significant differences between the LH and HL groups were only observed in the Construct Aggression and Deficient Narcissism categories. It may not always be easy to explain the differences between LH and HL. Previously used cutoffs have been relatively high [15–17], indicating that the diagnosis of HH was consistent and the application of lower cutoffs may make categorizing individuals into the LH and HL groups more difficult. According to the results of the principal component analysis, Intrusion-I, Avoidance-I, Hyperarousal-I, Intrusion-C, Avoidance-C, and Hyperarousal-C were all associated with PTSD severity, but Intrusion-I/Avoidance-I/Hyperarousal-I, Avoidance-C/Hyperarousal-C, and Intrusion-C demonstrated dissimilar associations. Thus, comparing Total-I and Total-C scores seemed to be insufficient, even when their averages were not identical in groups categorized by demographic and ethnocultural information [3].

To clarify the association between IES-R and CAPS scores and ISTA score, specific characteristics of the ISTA itself were also analyzed using correlation matrix and principal component analyses. It was not surprising that the analysis indicated the presence of differences between Destruct/Deficient and Construct because Construct consists of positive questions, whereas Destruct consists of negative questions, and Deficient questions assess lack of activity [13]. For example, in Aggression, the objective of the Construct items was to determine the level of active building within one’s own life, while Destruct was used to evaluate depreciation of other people, cynicism, and revenge, and Deficient was used to determine the occurrence of withdrawal into oneself and emotional emptiness. In Anxiety, the objective of the Construct items was to evaluate the level of general personality activation and realistic evaluation of danger, while Destruct was used to evaluate the occurrence of avoidance of new life experiences, and Deficient was used to determine the absence of protective functions. Those with a high Construct score may have low Destruct and/or Deficient scores and vice versa. The close and positive correlation identified between Destruct and Deficient also seems to be inherent.

However, when IES-R and CAPS scores were examined using a stepwise regression analysis with ISTA items used as determinants, a limited number of items were identified as significant. High Construct Aggression scores indicated a positive attitude toward life, and high Deficient Inner demarcation scores indicated the absence of a boundary between consciousness and unconsciousness, with individuals tending to place additional power in feelings, dreams, and fantasies [13]. It has been suggested that individuals with positive attitudes toward life and who are dependent upon feelings tended to have higher IES-R scores, but such a tendency was not observed in CAPS scores in this study. Instead, the negative effect of Construct Narcissism, or having a positive attitude toward one’s own life [13], was obvious in the CAPS scores. Other ISTA scores were identified to be associated with differences in IES-R and CAPS items. Hence, the dependence of IES-R and CAPS scores on the individual’s Ego structure differed under different conditions. Thus, the identification of a disagreement between IES-R and CAPS scores was inevitable.

The data used in the present study were obtained from a small ethnic group but suggested the existence of discrepancies between the IES-R and CAPS when used for PTSD diagnosis. Similar types of differences have also been previously reported in several situations [20–24]. To obtain more accurate and consistent PTSD diagnoses, the effects of personality traits, including those assessed using the ISTA in this study, on IES-R CAPS scores should also be examined, as the majority of studies have reported that individuals with negative attitudes toward life tended to develop PTSD [25–32]. Thus, individuals’ IES-R and CAPS scores were absolutely dependent upon his or her personality/ego structure. The ISTA is not always commonly used in Western countries but has been found to be comparable with the Minnesota Multiphasic Personality Inventory (MMPI) in different situations, thereby demonstrating the validity and reliability of these questionnaires [13]. However, the ISTA includes 220 questions, whereas the original MMPI consisted of 550 questions [14], and even its revised version, the MMPI-RF, contains 338 questions [33]. Such large numbers of questions are thought to be very difficult for subjects with PTSD to complete. The selection of an appropriate and limited number of questions is therefore important.

### Limitations of this study

The analysis was cross-sectional in nature and included indigenous people living in a very small and limited area where the effects of the outer environment were likely negligible. However, at the same time, their personality and ego structure might be affected by their specific ethnoculture [3]. Studies with the same purpose [25–32] have previously been conducted, but these studies assessed different ethnocultural populations and different hazards.

## Conclusion

To avoid the misdiagnosis of PTSD, usage of both the IES-R and the CAPS is required. Simultaneous application of personality and ego tests may be helpful in assigning diagnoses. Selection of an appropriate and limited number of questions that are related to those included on the MMPI and ISTA is important.

## Abbreviations

CAPS: Clinician-Administered PTSD Scale
HH: A group with high Total-I and high Total-C
HL: A group with high Total-I and low Total-C
IES-R: Impact of Event Scale-Revised
ISTA: Ego Structure Test by Ammon
LH: A group with low Total-I and high Total-C
LL: A group with low Total-I and low Total-C
PTSD: Post-traumatic stress disorder
Total-C: Total scores of CAPS
Total-I: Total scores of IES-R
